# The safety and efficacy of high-intensity interval training (HIIT) in post-stroke patients with moderate functional impairment: a systematic review and meta-analysis

**DOI:** 10.3389/fneur.2025.1695243

**Published:** 2025-11-19

**Authors:** Xueqi Wu, Dan Yang, Qiaochu Zhu, Yao Xiao, Hai Huang

**Affiliations:** 1 College of Acupuncture and Orthopedics, Hubei University of Chinese Medicine, Wuhan, China; 2 Rehabilitation Medicine Center/Tuina Department, Hubei Provincial Hospital of Traditional Chinese, Wuhan, China; 3 Hubei Shizhen Laboratory, Wuhan, China; 4 Hubei Key Laboratory of Theory and Application Research of Liver and Kidney in Traditional Chinese Medicine (Hubei Province Hospital of Traditional Chinese Medicine), Wuhan, China; 5 The First Clinical Medical School, Hubei University of Chinese Medicine, Wuhan, China

**Keywords:** post-stroke motor dysfunction, 6MWT, high-intensity interval training, rehabilitation, meta-analysis

## Abstract

**Objective:**

This study aimed to compare the efficacy and safety of high-intensity interval training (HIIT) versus conventional rehabilitation for improving lower limb function in post-stroke patients.

**Methods:**

A comprehensive literature search was conducted in PubMed, EMBASE, Web of Science, and Scopus from inception to January 2025. Only randomized controlled trials (RCTs) involving adults in post-stroke rehabilitation published in English were included, while grey literature was excluded. Standardized mean differences (SMDs) with 95% confidence intervals (CIs) were calculated. The primary outcomes were 6-min walk test (6MWT), Self-Selected Speed (SSS) and the Fastest Speed (FS). The secondary outcomes were peak oxygen uptake (Peak VO_2_) and SF-36 scores. The experimental group received high-intensity interval training (which involved robotic-assisted, cycling-based, or treadmill protocols targeting ≥60% of Peak VO₂), and the control group received standard care or regular exercise.

**Results:**

This meta-analysis included 10 studies. The results showed that high-intensity interval training has demonstrated significant improvements in walking ability and cardiopulmonary function compared with controls. High-intensity interval training had positive effects on 6MWT [*SMD* = 0.25, 95% *CI* (−0.01, 0.52)], SSS [*SMD* = 0.65, 95% *CI* (0.26, 1.03)], FS [*SMD* = 0.49, 95% *CI* (0.10, 0.88)], SF-36 scores [*SMD* = 0.67, 95% *CI* (0.04, 1.21)] and Peak VO₂ [*SMD* = 0.29, 95% *CI* (0.04, 0.54)] in stroke patients. According to the analysis, HIIT participants demonstrated better rehabilitation outcomes in walking capacity, cardiorespiratory function and quality of life.

**Conclusion:**

HIIT may be a safe and effective therapy for specific post-stroke patients, but more high-quality research is needed to confirm its efficacy and optimize protocols.

**Systematic review registration:**

This systematic review was registered in PROSPERO (Unique Identifier: CRD42025637166). The protocol can be accessed at: https://www.crd.york.ac.uk/PROSPERO/view/CRD42025637166.

## Introduction

Post-stroke motor dysfunction (PSD) refers to persistent motor impairment, which is caused by damage to the neural structures responsible for motor control (1). To delineate our study population, we focused on patients with moderate functional impairment, defined as a modified Rankin Scale (mRS) score of 3–4. Globally, approximately 50%–75% of stroke survivors experience varying degrees of motor dysfunction, such as hemiplegia or balance impairment (2). Among the various impairments following stroke, lower limb dyskinesia is one of the primary factors hindering recovery. According to the Copenhagen Stroke Study (3), 22% of stroke survivors are unable to walk, 14% require assistance to walk, and fewer than 10% regain adequate walking speed and endurance. These impairments impose significant caregiving, financial, and psychological burdens on the families of stroke survivors. In the United Kingdom, the societal cost of post-stroke rehabilitation is projected to reach £75 billion in 2035 (UK) (4). Thus, restoring independent walking ability is essential for promoting functional recovery and reducing caregiver burden.

Previous studies have demonstrated that rehabilitation within the first 3 months after stroke plays a critical role in long-term motor function recovery (5). However, conventional stroke rehabilitation programs often provide suboptimal duration or intensity (6). Moreover, most conventional rehabilitation therapies rely on specialized equipment, are constrained by environmental factors, and are costly, placing a heavy burden on patients’ families (7). In response, the 2023 AHA/ASA stroke rehabilitation guidelines strongly endorse high-intensity, task-specific training, high-intensity interval training (HIIT) has emerged as a potential alternative (8).

The efficacy of high-intensity interval training (HIIT) in stroke recovery is supported by its multi-level physiological effects. Neurologically, HIIT promotes neuroplasticity and cortical reorganization by upregulating BDNF/TrkB signaling and modulating inhibitory circuits (9, 10). Systemically, it enhances cardiorespiratory fitness and cerebral blood flow, thereby improving the metabolic support for brain repair and collectively driving improvements in motor function.

Recent studies (11–13) have shown that high-intensity interval training (HIIT) is an effective intervention for patients in the 3–6 month recovery phase after stroke. HIIT is characterized by alternating bouts of higher-intensity exercise (≥60%–80% peak VO₂) and lower-intensity exercise (40%–50% for recovery). Compared to conventional rehabilitation, it can increase muscle strength, reduce spasticity, enhance endurance, and improve cardiopulmonary function, thereby supporting patients in daily life after recovery. Furthermore, the alternating structure of HIIT makes it less monotonous and more engaging than continuous exercise, reducing dropout rates by up to 30% and improving overall adherence (14).

However, some studies have suggested that high-intensity interval training (HIIT) may cause a rapid increase in heart rate, potentially leading to discomfort or safety concerns (15–19). Three of the original studies reported negative or non-beneficial outcomes in certain functional indicators, which may confound clinical decision-making. Therefore, this systematic review and meta-analysis aimed to comprehensively evaluate the safety and efficacy of HIIT in post-stroke rehabilitation to inform clinical practice.

## Methods

This meta-analysis adhered to the Preferred Reporting Items for Systematic Reviews and Meta-Analyses (PRISMA) guidelines (20) and was prospectively registered on the PROSPERO platform on January 22, 2025 (CRD42025637166). Since this study analyzes previously published data, ethical review is not required.

### Search strategy

We systematically searched the following electronic databases from inception to January 2025: PubMed, EMBASE, Web of Science, Scopus, and Ovid/Medline. Our search employed MeSH and free-text terms, using the Boolean operator OR to encompass synonyms for “Stroke,” “High-Intensity Interval Training,” and “Randomized Controlled Trial,” and the operator AND to ensure results combined all three core concepts. The complete search strategy is available in the Supplementary Material S3.

### Inclusion exclusion criteria

After completing the search, studies were screened based on the following inclusion and exclusion criteria: (1) Study design: only randomized controlled trials (RCTs) were included, (2) Participants: adults (≥18 years old) in the rehabilitation phase of acute, subacute, or chronic stroke, (3) The HIIT intervention had to report intensity, duration, and frequency, and could include devices such as treadmills or cycle ergometers, (4) The standard for low-to-moderate intensity continuous training is a peak oxygen uptake of <50%, or the provision of routine care or no intervention, (5) Outcomes: studies must report at least one of the following outcome measures—6-min walk test (6MWT), self-selected walking speed, fastest walking speed, peak VO₂, or SF-36 scores, studies with incomplete or unclear outcome data were excluded.

Exclusion criteria were defined as follows: (1) Studies not published in English, (2) Non-English studies, conference abstracts, case reports, and studies with missing or incomplete data were excluded.

### Risk of bias

Two blinded independent researchers (XQ and YD) used the Cochrane Risk of Bias Tool [ROB 2.0 (Cochrane Collaboration, 2019, Odense, DK)] to evaluate the quality of included studies from seven dimensions: (1) Bias due to the randomization process, (2) Bias due to the allocation process, (3) Bias due to blinding deficiencies, (4) Bias due to the assessment of outcomes, (5) Bias due to follow-up, (6) Bias due to reporting of results, (7) Other biases. Any disagreements were resolved through consultation with a third reviewer.

### Quality of evidence

Evidence quality was assessed using the GRADE framework, categorizing certainty as high, moderate, low, or very low based on risk of bias, inconsistency, indirectness, imprecision, and publication bias. All assessments were conducted using GRADEpro GDT (McMaster University, Hamilton, Canada).

### Data extraction

Data extraction was performed in accordance with the Cochrane Handbook for Systematic Reviews of Interventions. A standardized Excel-based data extraction form was developed based on the Handbook. Prior to formal data extraction, the form was piloted on two randomly selected included studies to ensure clarity and consistency between reviewers. The first reviewer (XQ) extracted data from all eligible studies using the refined form, and the second reviewer (YD) independently verified the accuracy of the extracted data. Any discrepancies were resolved through discussion and consensus.

Data included study characteristics (author, year, sample size, mean age, time since stroke), intervention details (intensity, duration), and outcomes (6MWT, SSS, FS, peak VO₂, SF-36). If a study reported both pre- and post-intervention means and standard deviations (SDs), the mean difference and corresponding SD were calculated using the formulas recommended in the Cochrane Handbook for Systematic Reviews of Interventions. Specifically, when SDs were not directly available, they were imputed from 95% confidence intervals using the prescribed method.

### Statistical analysis

All statistical analyses were meta-analyzed using STATA 18.0 and RevMan 5.3.4. For continuous variables, standardized mean difference (SMD) and 95% confidence interval (CI) were used for analysis. Heterogeneity was quantified using *I*^2^, categorized as low (<25%), moderate (25%–75%), or high (>75%). A fixed-effects model was applied when heterogeneity was low (*I*^2^ ≤ 50%); otherwise, a random-effects model was used to account for potential clinical variability among the trials. To evaluate the robustness of the results, sensitivity analyses were conducted. Publication bias was assessed using funnel plots and Egger’s test, with statistical significance set at *p* < 0.10 for the latter.

## Results

### Study screening, selection, and evaluation

**Figure 1:**
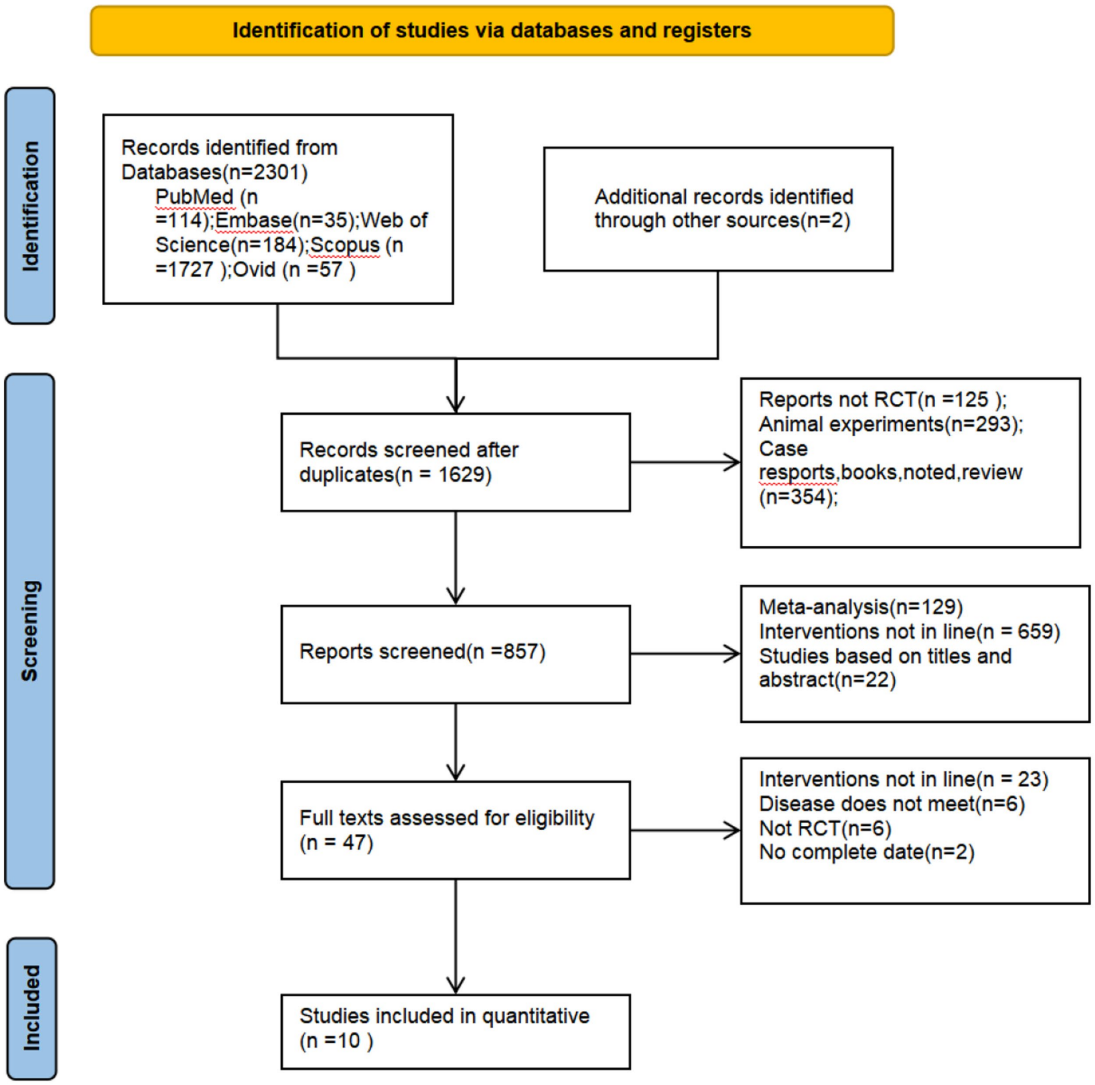
Flow diagram of the selection process.

As of January 2025, a preliminary total of 1,627 documents were identified through searches of five databases. After removing 674 duplicates, the remaining records were screened by title, abstract, and full text according to the inclusion and exclusion criteria. Additionally, we employed a backward citation tracing approach, screening the reference lists of included studies to identify two eligible studies. Ultimately, 10 studies were included in the quantitative synthesis. Figure 1 shows the PRISMA flow diagram of study selection.

### Studies included in the systematic review

A total of 10 studies included 370 participants with the weighted mean age across all included studies was 61.1 ± 11.8 years. The mean time since stroke onset ranged from 17 to 88 months. Among them, 186 participants received high-intensity interval training (HIIT), while 184 received moderate-intensity continuous training (MICT) or usual care.

Interventions included treadmill-based (2, 21–25), robot-assisted (22), and cycling protocols (26) targeting 60%–85% of HRR or VO₂peak. The included studies reported exercise intensities ranging from 60% to 85% of HRR or VO₂peak, with total session durations ranging from 25 to 50 min (most commonly 30–40 min). Training frequency is 2–5 sessions per week, lasting 4–24 weeks (Table 1).

**Table 1:** Characteristics of included studies.

Study	Sample size (exp/con) (exp/con)	Mean age (exp/con)	DISEASE duration (exp/con)	Intervention/control	Exercise intensity	Course of treatment (exp/con)	Outcomes	Follow-up duration
Lapointe et al. (14)	15/12	71.8 ± 9.9/69.6 ± 10.7	39.3 ± 61.0 mo	HIIT/routine care	95% of PPO	24 weeks	SBP/HAD/ Peak VO_2_/PPO	48 weeks
Hsu et al. (26)	10/13	58.5 ± 12.16/53.1 ± 9.65	38.5 ± 27.12/28.8 ± 42.01 mo	HIIT/MICT	80% of peak VO₂	12 weeks	Peak VO_2_/AV O2diff/Δ [HHb]/BDNF	no mentioned
Hornby et al. (25)	17/11	57 ± 9.72/66 ± 11.9	42 ± 45.71/20 ± 19.72 mo	HIIT(AIT+HIT)/HIT+normoxia	75% of HRR	5 weeks	Peak VO_2_/SSS/FS/6MWT	no mentioned
Yu et al. (24)	14/16	61.86 ± 11.33/46.94 ± 15.61	511.07 ± 292.91/475.31 ± 411.42 days	HIIT(exowalk60min)/exowalk30min	No mentioned	2 weeks/4 weeks	FAC/6MWT	no mentioned
Lee et al. (27)	12/12/12/12	60.5 ± 10.6/65.3 ± 6	57.0 ± 54.2 mo	HIIT(Cycling)/sham cycling+sham PRT	70% of peak VO₂	12 weeks	Peak VO_2_/PPO/6MWT	no mentioned
Hornby et al. (25)	12/17/15	52 ± 13/57 ± 12	3.2 ± 1.8/3.7 ± 1.8 mo	HIIT/routine care	70–80% of HRR	10 weeks	SF-36/SSS	8-12 weeks
Aidar et al. (29)	11/11	51.7 ± 8/52.5 ± 7.7	No mentioned	HIIT/routin	70–80% of HRR	12 weeks	SF-36	no mentioned
Boyne et al. (21)	27/28	63.8 ± 9.9/61.5 ± 9.9	2.7 ± 1.4/2.2 ± 1.2 year	HIIT/MAT	60% of HRR	12 weeks	VO_2_max/SSS/FS/6MWT	12 weeks
Do et al. (22)	11/11	61.8 ± 7.3/63.5 ± 8.1	88.0 ± 81.5/81.5 ± 52.4 mo	HIIT(RATW)/control	70% of HRR	8 weeks	VO_2_max/10MWT/FAC	8 weeks
Moncion et al. (23)	42/40	65.4 ± 8.9/64.4 ± 9.7	1.9 ± 1.3/1.7 ± 1.3 year	HIIT/MICT	70–80% of HRR	20 weeks	VO_2_max/6MWT/SBP	8 weeks

VO_2_max, maximal oxygen consumption; Peak VO_2_, peak oxygen uptake; HRR, Heart Rate Reserve; SBP, systolic blood pressure; HAD, hospital anxiety and depression scale; PPO, peak power output; AVO_2_diff, arteriovenous O_2_ difference; BDNF, brain-derived neurotrophic factor; Δ[HHb], deoxyhemoglobin; SSS, self-selected speed; FS, fastest speed; 6MWT, 6-min walk test; SF-36, Medical Outcomes Short Form −36 questions; 10MWT, 10-meter walk test; FAC, functional ambulatory catego.

High intensity: Defined as meeting any of the following criteria: ≥70% heart rate reserve; ≥70% peak oxygen uptake; ≥95% peak power output (HIIT mode); Significantly higher single-session duration/dose compared to the control group while maintaining equivalent total training volume. Moderate/Low Intensity: Defined as: Approximately 30%–60% of heart rate reserve; Approximately 60% of peak oxygen uptake; Routine care/home exercise; Sham intervention (Table 2).

**Table 2:** Summary of operational definitions for high-intensity and moderate/low-intensity training in the included studies.

Study	HIIT intensity definition	Control intensity definition
Lapointe et al. (14)	95% of PPO interspersed with a 60-s recovery	Usual care without any additional physical activity
Hsu et al. (26)	80% of peak VO₂ low-intensity recovery periods at 40% VO_2_ peak	60% of peak VO₂
Hornby et al. (25)	75% of HRR	Constant oxygen exposure
Yu et al. (24)	Exowalk/60 min	Exowalk/30 min
Lee et al. (27)	70% of peak VO₂	Sham cycling
Hornby et al. (25)	70–80% of HRR	30–40% of HRR
Aidar et al. (29)	Structured strength training	No strength training
Boyne et al. (21)	60% of HRR	40% ± 5% of HRR
Do et al. (22)	70% of HRR	Routine care
Moncion et al. (23)	80% of HRR	40%–59% of HRR

### Quality assessment and risk of bias

**Figure 2:**
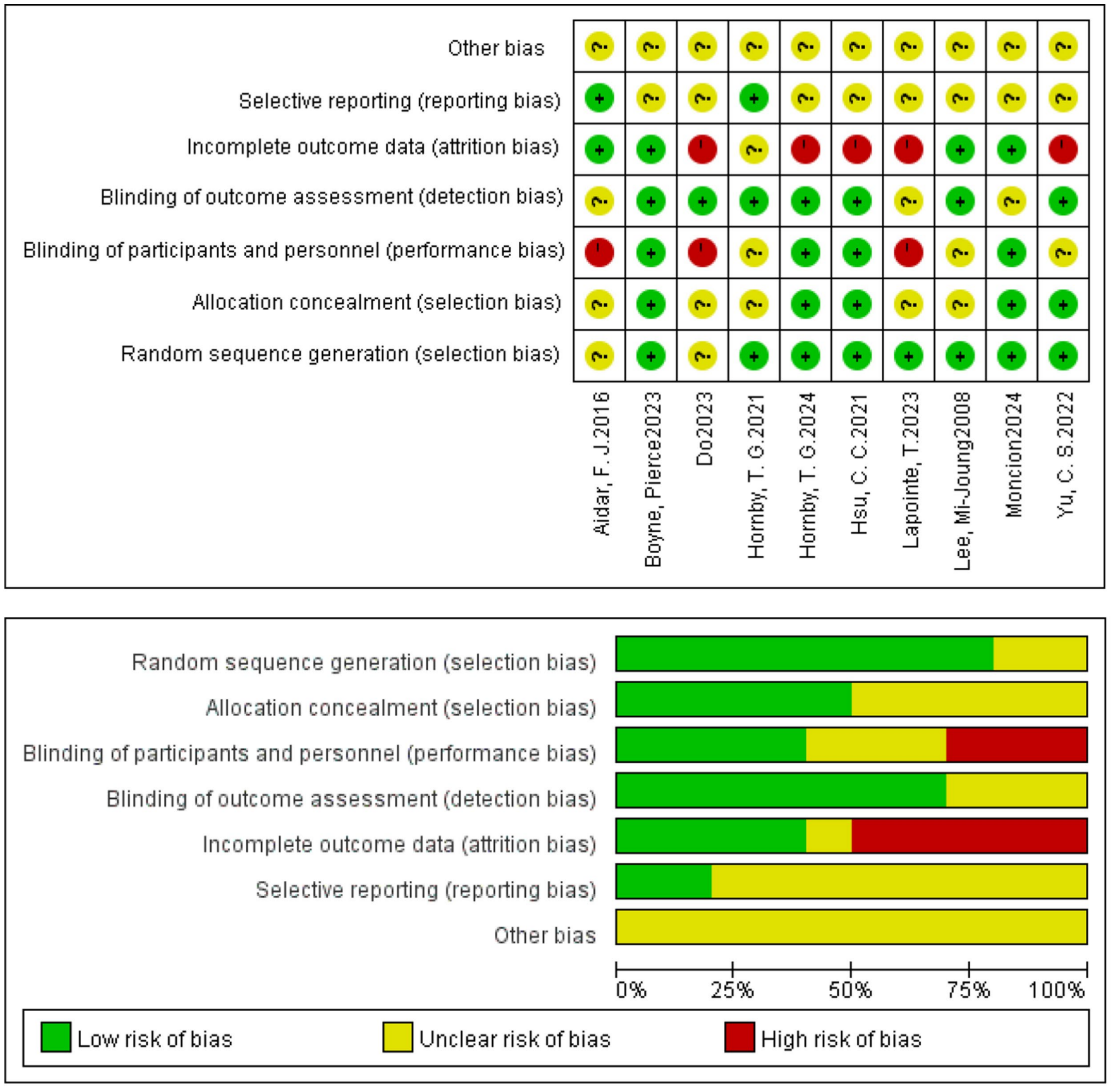
Risk of bias summary graph for included studies.

Risk of bias was assessed across seven Cochrane domains using RevMan 5.4. Overall, the included studies generally performed well in random sequence generation but exhibited a high risk of bias in allocation concealment and blinding (Figure 2; Table 3).

**Table 3:** Quality assessment and risk of bias.

Study	Randomization process	Allocation process	Blinding deficiencies	Assessment of outcomes	Follow-up	Reporting of results	Other bias
Lapointe et al. (14)	Low	Low	High	Low	Low	Unclear	Unclear
Hsu et al. (26)	Low	Low	Low	Low	Low	Unclear	Unclear
Hornby et al. (25)	Low	Low	Low	Low	Low	Unclear	Unclear
Yu et al. (24)	Low	Low	Unclear	Low	Low	Unclear	Unclear
Lee et al. (27)	Low	Unclear	Unclear	Low	Low	Unclear	Unclear
Hornby et al. (25)	Low	Unclear	Unclear	Low	Low	Low	Unclear
Aidar et al. (29)	Unclear	Unclear	High	Unclear	Low	Low	Unclear
Boyne et al. (21)	Low	Low	Low	Low	Low	Unclear	Unclear
Do et al. (22)	Unclear	Unclear	High	Low	Low	Unclear	Unclear
Moncion et al. (23)	Low	Low	Low	Unclear	Low	Unclear	Unclear

## Meta-analysis of results

### Primary outcomes

#### 6MWT (6-min walk test)

**Figure 3:**
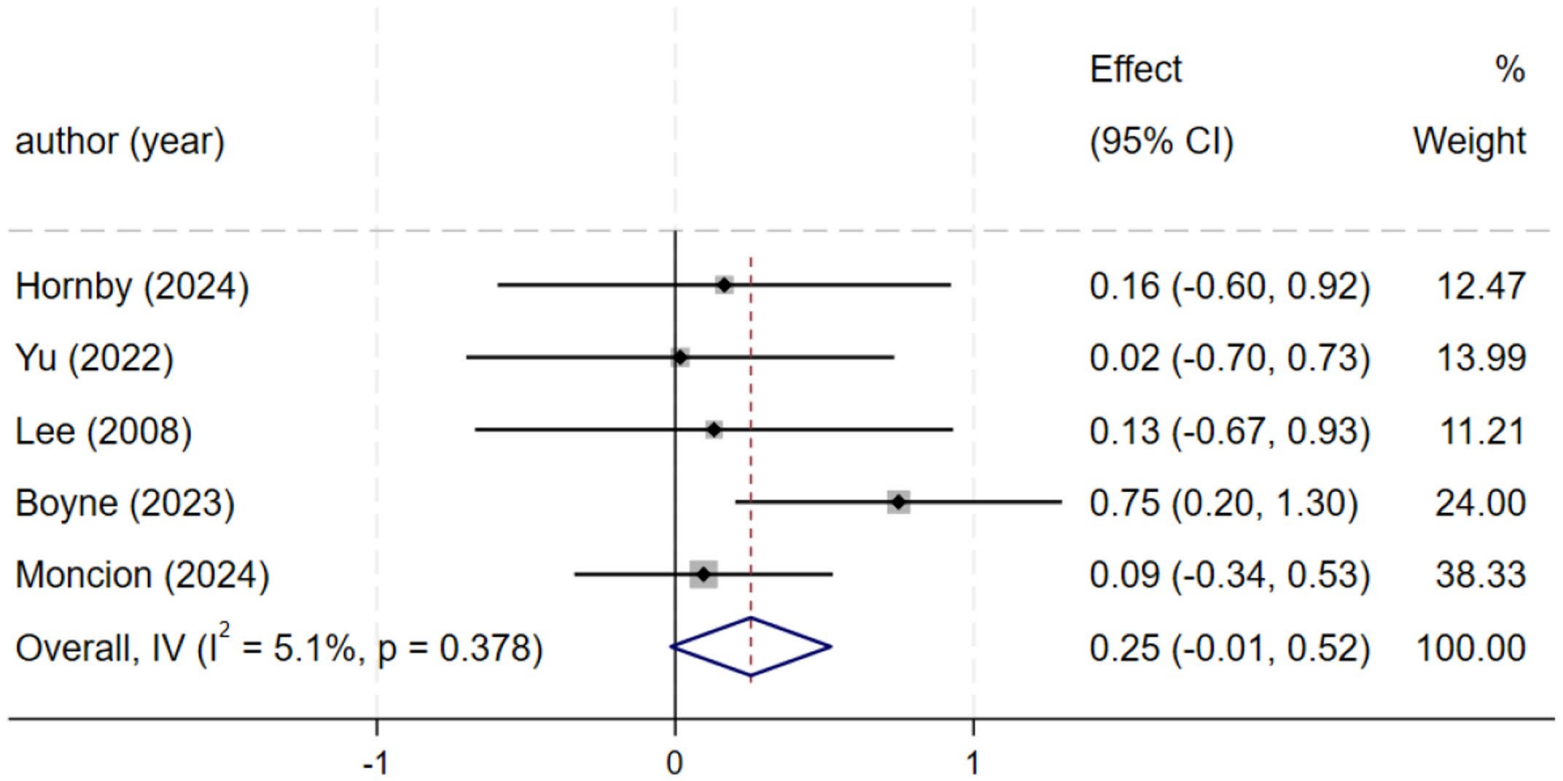
Forest plot of 6MWT.

Five studies (2, 21, 23, 24, 27), involving a total of 219 patients, reported data on the 6-min walk test (6MWT). A fixed-effects model (*I*^2^ = 5.1%, *p* = 0.378) indicated no significant difference between HIIT and MICT. The pooled results demonstrated a small, non-significant improvement in 6MWT distance favoring HIIT over MICT [SMD = 0.25, 95% CI (−0.01, 0.52), Figure 3]. Although this result did not reach statistical significance, the point estimate supports the possibility of a positive trend. This uncertainty may be attributable to the limited sample size. This finding aligns with Luo et al. (28), who reported a significant improvement in walking capacity, lending support to the potential benefit of HIIT (28). Consequently, further large-scale RCTs are warranted to confirm the effect of HIIT on 6MWT performance.

#### SSS (speed of self-selection)

**Figure 4:**
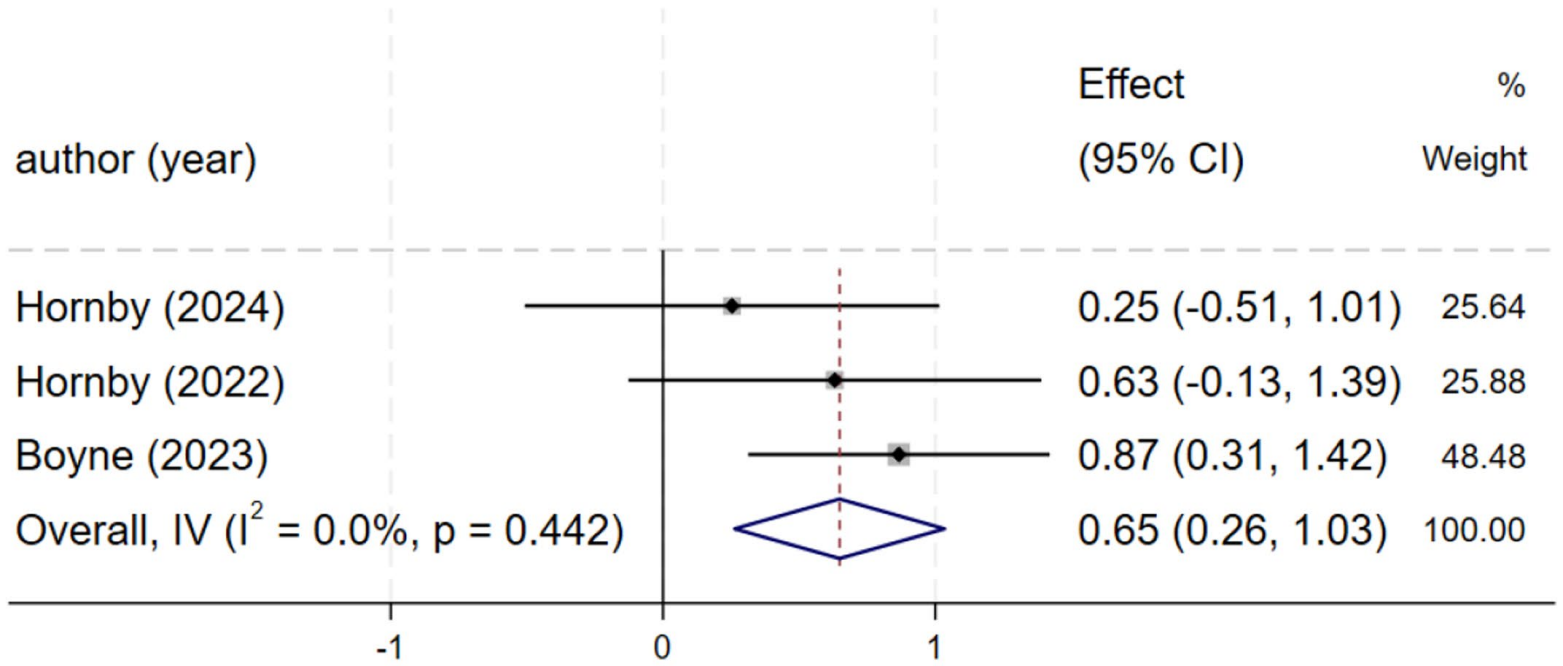
Forest plot of SSS.

Three studies (2, 21, 25) reported self-selected walking speed. Homogeneity was observed among these studies (*I*^2^ = 0.0%, *p* = 0.442). Therefore, a fixed-effects model was chosen for the analysis of this outcome. Compared with the control group, HIIT demonstrated a positive effect in improving self-selected speed, with a moderate effect size [SMD = 0.65, 95% CI (0.26, 1.04), Figure 4] that suggests potential clinical relevance for functional mobility in stroke patients.

#### FS (fastest speed)

**Figure 5:**
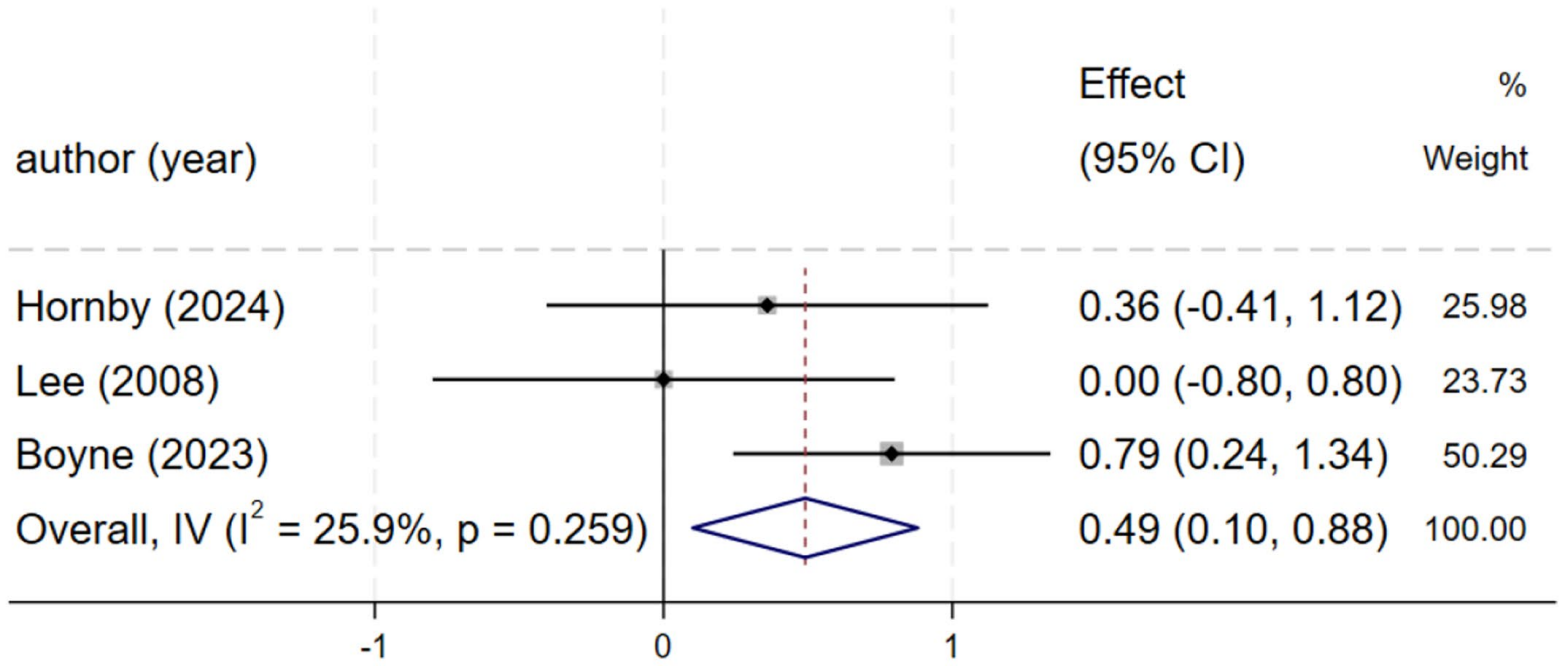
Forest plot of FS.

Three studies (2, 21, 27) reporting fastest speed demonstrated low heterogeneity (*I*^2^ = 25.9%, *p* = 0.259), justifying a fixed-effects model. The meta-analysis revealed that HIIT significantly increased fastest speed compared to control conditions, with a standardized mean difference [SMD = 0.49, 95% CI (0.10, 0.88), Figure 5].

### Secondary endings

#### Peak VO_2_ value

**Figure 6:**
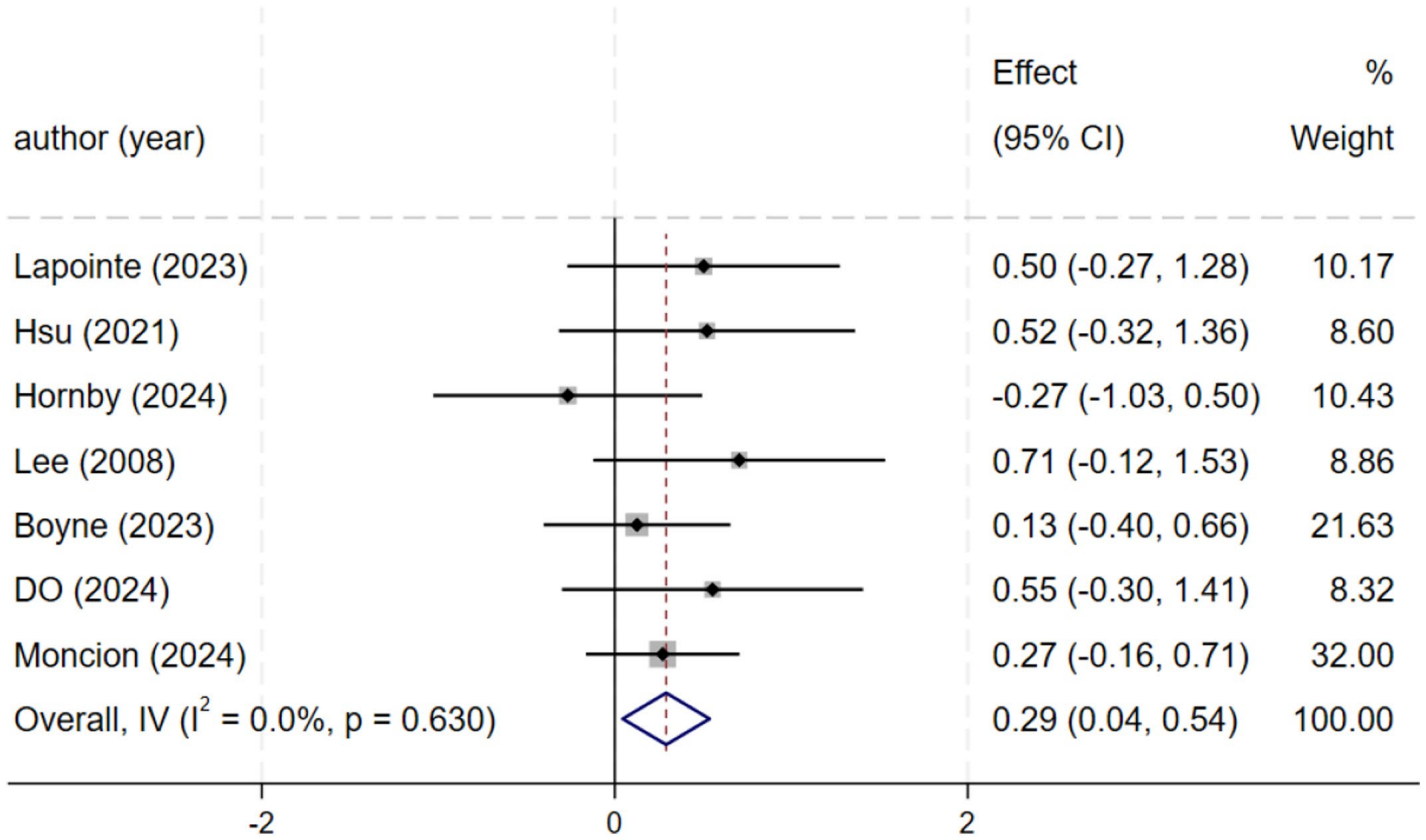
Forest plot of VO_2_.

Seven studies (*n* = 261) demonstrated significant improvements in peak VO₂ [SMD = 0.29, 95% CI (0.04, 0.54); *I*^2^ = 0%, *p* = 0.630]. Although peak VO₂ was not a primary outcome, its improvement indicates enhanced cardiopulmonary capacity, supporting motor recovery (Figure 6).

#### SF-36

**Figure 7:**
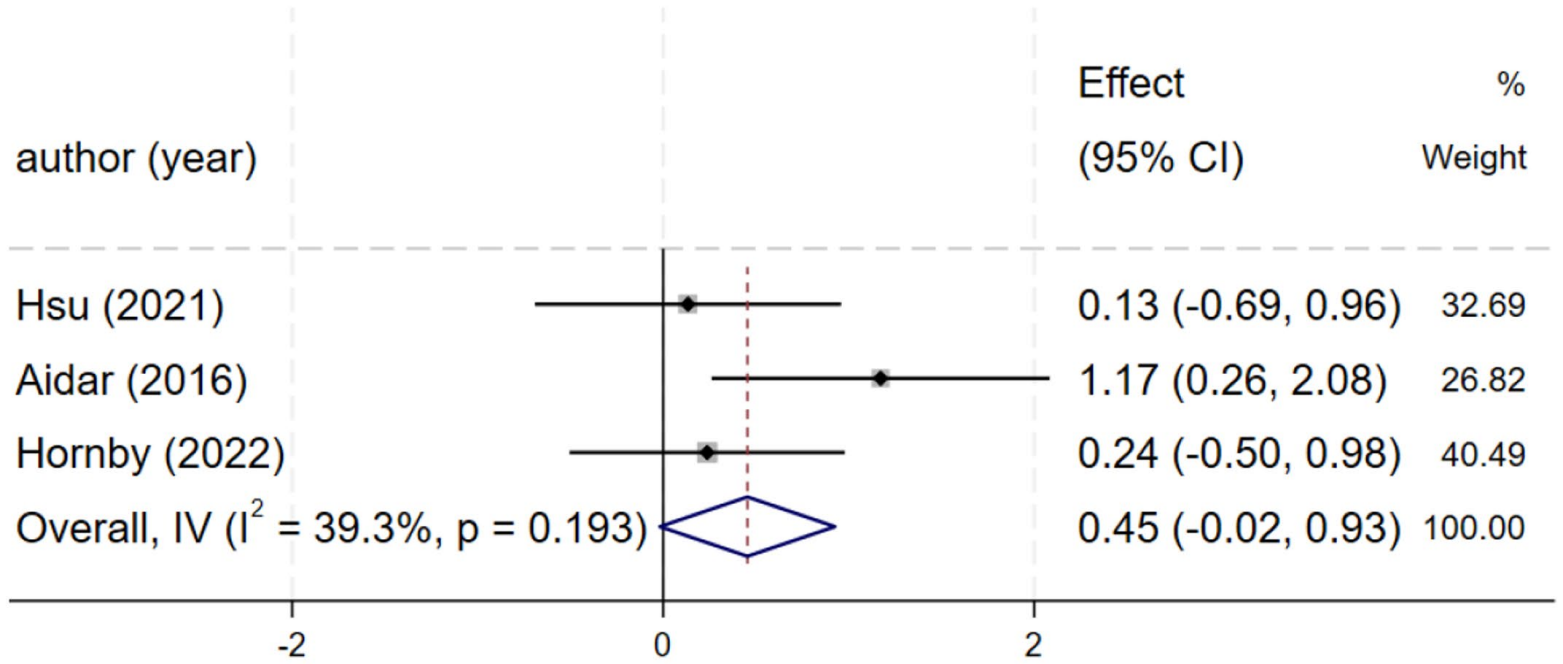
Forest plot of SF-36.

Three studies (25, 26, 29) reporting SF-36 scores demonstrated moderate heterogeneity (*I*^2^ = 39.0%, *p* = 0.194), and a fixed-effects model was consequently applied. The meta-analysis found a non-significant effect [SMD = 0.46, 95% CI (−0.02, 0.94), Figure 7]. Due to the wide confidence interval that spans the zero value, the effect estimate is subject to uncertainty. This finding is inconclusive regarding quality of life, and the observed effect may be a chance finding due to the limited statistical power of the included studies.

### Publication bias and sensitivity analysis

**Figure 8:**
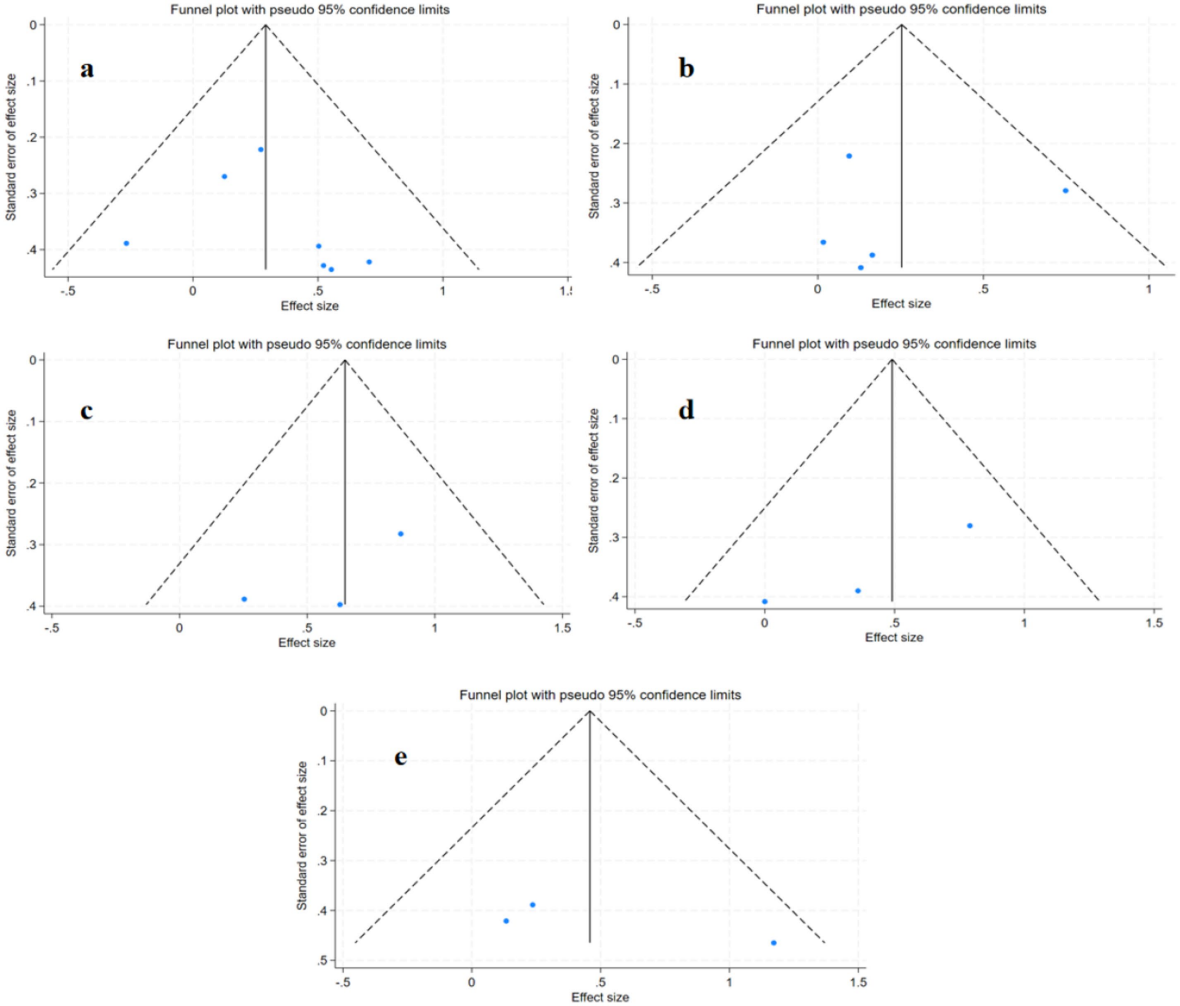
Publication bias plots for different outcome measures: **(a)** VO₂; **(b)** 6MWT; **(c)** SSS; **(d)** FS; **(e)** SF-36.

Publication bias was assessed using funnel plots and Egger’s test. The results showed no significant publication bias for any outcome. However, the limited number of studies reduces the power of funnel plot asymmetry tests. (Figure 8): primary outcome 6MWT (*p* = 0.857, Figure 8b), SSS (*p* = 0.391, Figure 8c), FS (*p* = 0.185, Figure 8d), secondary outcomes VO_2_ (*p* = 0.420, Figure 8a), SF-36 (*p* = 0.360, Figure 8e).

**Figure 9:**
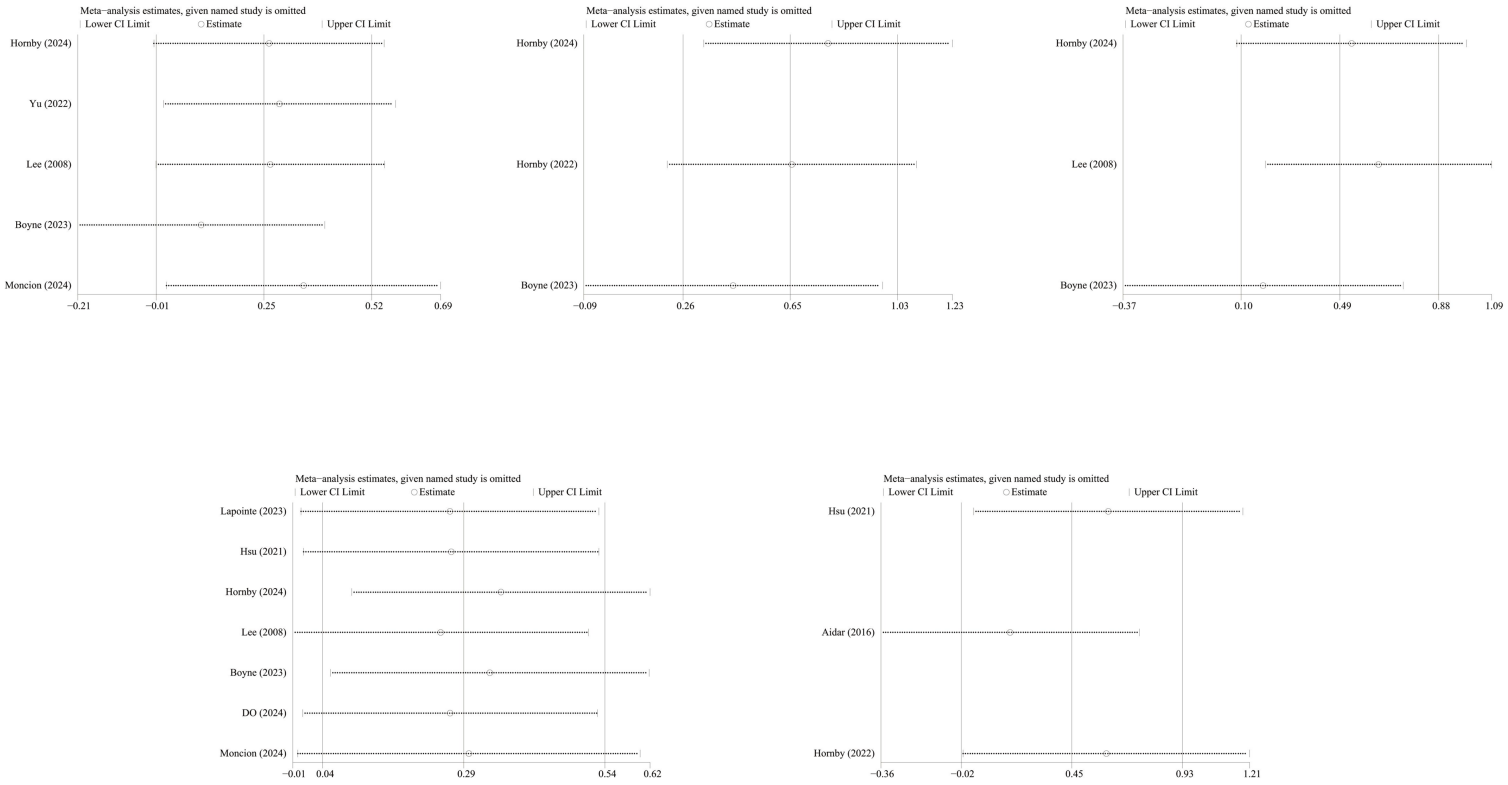
Sensitivity analysis.

To assess the robustness of the results, we conducted a sensitivity analysis by sequentially excluding individual studies. After excluding the study by Boyne et al. (21), the combined effect size for the 6-min walk test and functional status showed a significant decline (Figure 9).

### Adverse events, dropout rate, and compliance

The safety and compliance data from the included studies are summarized in Table 4. Most studies did not report serious adverse events associated with HIIT interventions. The dropout rates ranged from 0% to 30% in the intervention groups and from 0% to 25% in the control groups. Common reasons for discontinuation included changes in participants’ health status and external factors such as the COVID-19 pandemic. Overall compliance rates were generally high in the intervention groups, ranging from 77% to 99%.

**Table 4:** Summary of safety and compliance for interventions.

Study	Total Participants (*n*)	Adverse events in the intervention group	Adverse events in the control group	Overall shedding rate (exp/con)	Primary causes of shedding	Compliance (%)
Lapointe et al. (14)	36	No serious adverse events	No serious adverse events	21%/29%	Lack of interest (*n* = 6) Change in medical condition (*n* = 3)	HIIT group (77%)
Hsu et al. (26)	28	No serious adverse events	No serious adverse events	23%/13.3%	Recurrent stroke (*n* = 2) Unstable BP (*n* = 1) Hernia surgery (*n* = 1) Incomplete ex. (*n* = 1)	No mentioned
Hornby et al. (25)	35	One serious adverse event was observed following AIH exposure	No serious adverse events	15%/26.7%	Personal reasons (*n* = 5) Taking banned substances (*n* = 1) Dizziness reaction (*n* = 1) Personal reasons	No mentioned
Yu et al. (24)	36	No serious adverse events	No serious adverse events	22.2%/11.1%	FAC was level 1 (*n* = 2) FAC was level 6 (*n* = 4)	No mentioned
Lee et al. (27)	25	No serious adverse events	No serious adverse events	7.6%/0%	Changes in their health status (*n* = 1)	No mentioned
Hornby et al. (25)	29	No serious adverse events	No serious adverse events	0%/0%	No detachment has occurred	No mentioned
Aidar et al. (29)	27	Three patients dropped out due to personal circumstances.	Two patients dropped out due to personal circumstances.	0%/15.3%	Personal reasons (*n* = 5)	HIIT group (94%) Control group (No mentioned)
Boyne et al. (21)	55	No serious adverse events	No serious adverse events	30%/18%	Participants voluntarily withdrew (*n* = 7) Back pain (*n* = 1) Recurrent hamstring strain (*n* = 1) COVID-19 (*n* = 4)	HIIT group (82.3%) Control group (86.8%)
Do et al. (22)	24	No serious adverse events	No serious adverse events	8.3%/8.3%	Unknown reason (*n* = 2)	No mentioned
Moncion et al. (23)	82	No serious adverse events	No serious adverse events	21.4%/32,5%	Medical conditions (*n* = 9) COVID-19 (*n* = 6) Transportation issues (*n* = 2) Return to work (*n* = 2) Other rehabilitation (*n* = 1) Preference against intervention plan (*n* = 1) Unknown reasons (*n* = 1) Missing (*n* = 12)	HIIT group (99%) Control group (99%)

### GRADE analysis for the certainty of the evidence

**Figure 10:**
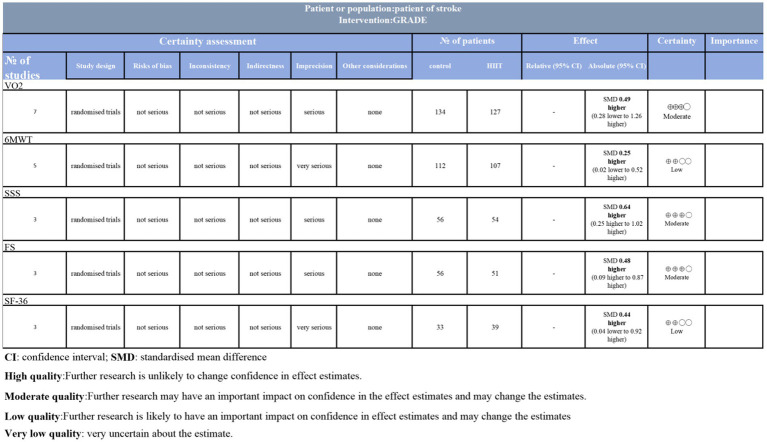
GRADE analysis and certainty of evidence.

Although the included randomized controlled trials (RCTs) are considered the highest level of evidence, the quality of the results still requires cautious interpretation. Certainty for VO₂, SSS, and FS outcomes was rated as moderate due to imprecision, while 6MWT and SF-36 outcomes were downgraded to low due to heterogeneity (Figure 10).

## Discussion

This meta-analysis of 10 RCTs supports the efficacy and safety of HIIT for improving functional recovery in post-stroke patients compared with conventional rehabilitation (30).

As a key indicator of walking ability, gait speed was assessed under two conditions (11, 30): self-selected speed, which reflects a sustainable pace chosen to avoid fatigue, and fastest speed, which challenges physiological limits to enhance cardiopulmonary function and muscle metabolism (31).

Our study demonstrates that the results possess significant clinical significance (*p* < 0.05). Furthermore, improvements in SSS [MD = 0.13 m/s, 95% CI (0.06, 0.20)] and FS [MD = 0.15 m/s, 95% CI (0.05, 0.26)] both exceeded the recognized minimum clinically important difference (MCID) threshold (0.10 m/s) (32). Compared to MICT, HIIT provided significantly greater and clinically relevant improvements in both self-selected and fastest gait speeds. This consistent advantage across different measures of walking ability reinforces the findings of prior studies (30). Collectively, the evidence suggests that HIIT should be considered an effective component of stroke rehabilitation programs.

Although the improvement of HIIT on the 6MWT did not reach statistical significance, the observed trend may hold clinical significance and warrants further investigation. Compared to moderate-intensity continuous training (MICT), high-intensity interval training (HIIT) demonstrated significant improvements in walking distance, indicating its potential clinical value. Combined with prior evidence suggesting HIIT is safer and more compliant (33, 34), it should be considered in rehabilitation practice and subjected to in-depth research.

*VO₂peak* remains an important outcome to consider. Although it does not directly reflect lower-limb functional recovery, walking endurance—an essential component of walking ability—largely depends on cardiopulmonary capacity (33, 35). Studies have demonstrated a positive correlation between *6MWT* distance and *VO₂peak*, indicating that improvements in *VO₂peak* can translate into greater walking endurance, a finding consistent with previous research (11, 30, 36, 37).

Although the SF-36 score did not reach statistical significance, it still indicated a positive impact on patients’ quality of life. Recovery of limb function not only reduces patients’ own burden but also alleviates stress on their families, thereby improving overall quality of life (38, 39). These findings are consistent with those reported by Reed et al. (33).

Although the results indicate that the intervention group showed overall positive efficacy, it is still necessary to assess the robustness of these findings and explore the sources of heterogeneity.

### Robustness of research findings and sources of heterogeneity

Our sensitivity analysis identified that the robustness of the combined results for the 6-min walk test and functional status is low. After in-depth comparison, we hypothesize that heterogeneity likely stems from two sources: first, Boyne et al.’s study included patients with poorer baseline average walking ability, setting a higher “ceiling” for physiological improvement and potentially leading to greater functional gains following intervention.

Notably, the core training protocol employed by Boyne et al. was high-intensity interval training (HIIT)—walking at maximum safe speed for 30 s followed by 30–60 s of rest. In contrast, Lee et al. (27) separated training components by establishing a sham training control group. While this approach effectively isolated variables, the neuromuscular and metabolic stimulation intensity it provided was significantly lower than the protocol designed for functional impairment (walking) in Boyne et al.’s study. The superiority of this protocol in functional relevance and overall stimulation intensity likely explains its outstanding clinical outcomes.

Therefore, the heterogeneity of existing evidence does not negate the efficacy of HIIT but rather reveals that its effectiveness depends on highly specific training protocols and patient populations with improvement potential. For its clinical application, in addition to efficacy, safety and acceptability are of paramount importance.

### Clinical feasibility of HIIT: safety and compliance analysis

Based on existing research, HIIT demonstrates good safety for stroke patients. The vast majority of studies reported no intervention-related serious adverse events, indicating low risk when HIIT is implemented under normal conditions.

Regarding tolerability, while dropout rates varied across studies, no consistent pattern of higher rates in intervention versus control groups emerged. High dropout rates were predominantly linked to non-directly related factors such as changes in individual health status or COVID-19 impacts, rather than training intensity itself, suggesting overall acceptable tolerability.

Adherence data showed that compliance rates in HIIT groups were generally high (e.g., 77% to 99%), indicating patients’ ability to persist with high-intensity training regimens. However, some studies did not report this data, which should be improved in future research.

It is important to note that, based on the GRADE assessment and the low robustness in sensitivity analyses, the overall certainty of evidence regarding the efficacy of HIIT is low to moderate. Therefore, any clinical recommendations based on these findings should be treated with caution.

### Limitations

This study has several limitations. First, the included trials varied in study design, participant characteristics, interventions, and outcome measures, which may have influenced pooled effect estimates. Some studies exhibit a high risk of bias in allocation concealment and blinding (14, 25, 27, 29), The absence of blinding may lead participants and researchers to exhibit a halo effect on subjective outcome measures (such as functional scores), thereby increasing their tendency to report positive results. At the same time, inadequate allocation concealment may lead to researcher bias during patient inclusion, thereby undermining the effectiveness of randomization.

Therefore, subsequent studies should prioritize allocation concealment and ensure at least evaluator blinding, while increasingly utilizing objective physiological indicators (such as muscle strength testing and grip strength testing) to report patient outcomes.

Second, the relatively small cumulative sample size (*n* = 370) may have limited the statistical power to detect small but meaningful effects. Future research should focus on recruiting larger sample sizes to enhance statistical power, ensuring the ability to detect the smallest clinically meaningful differences.

Third, this study included only articles published in English, which have excluding non-English studies may bias results toward positive findings. Therefore, future meta-analyses should include a comprehensive, multilingual literature search to verify whether the positive effects of HIIT hold across a more diverse evidence base.

Fourth, variations in the definition of “intensity” between intervention and control groups—such as the use of different physiological metrics (e.g., %HRR vs. % VO₂peak), as well as differences in rehabilitation standards across studies, may have affected the objectivity and reliability of the analysis. Future studies should adopt standardized operational definitions of exercise intensity (e.g., consistently using %HRR) and provide detailed descriptions of control interventions. This will enhance the comparability of results across trials and strengthen the evidence base.

Fifth, transformations were applied to estimate means and standard deviations from studies that reported only medians and interquartile ranges (IQRs), which may have introduced additional heterogeneity and measurement error. Finally, most included studies recruited patients with mild to moderate post-stroke impairments, without considering individuals with other cardiovascular conditions.

Future research should adopt larger-scale, multicenter randomized controlled trials with long-term follow-up, enrolling patient populations with varying degrees of impairment. These studies should also explore neurophysiological indicators during rehabilitation (e.g., BDNF, cerebral perfusion, electromyographic activity). Furthermore, conducting cost-effectiveness and feasibility studies will be crucial to facilitate the translation of HIIT technology into routine clinical practice.

## Conclusion

In conclusion, current evidence suggests this therapy may represent a safe and effective treatment strategy for specific post-stroke patients, but further high-quality studies are needed to validate these findings and optimize training parameters.

## Data Availability

The original contributions presented in the study are included in the article/Supplementary material, further inquiries can be directed to the corresponding authors.
