# Supplementary material for: The safety and efficacy of high-intensity interval training (HIIT) in post-stroke patients with moderate functional impairment: a systematic review and meta-analysis
Source: Front Neurol. 2025 Nov 19;16:1695243. doi: 10.3389/fneur.2025.1695243 (PMC12675936; doi:10.3389/fneur.2025.1695243)
Supplement: Supplementary file 1 [file Data_Sheet_1.docx]

**Appendix 1**

Appendix 1 contains search terms specific to each database to ensure reproducibility

Search strategy of PubMed

| Search  number | Query | Results |
| --- | --- | --- |
| #1 | **"Stroke"[Mesh]** | 187362 |
| #2 | **(((((Cerebral Strokes[Title/Abstract]) OR (Acute Stroke[Title/Abstract])) OR (Cerebral embolism[Title/Abstract])) OR (Cerebral infarction[Title/Abstract])) OR (Ischemic stroke[Title/Abstract])) OR (Acute Cerebrovascular Accident[Title/Abstract])** | 106139 |
| #3 | #1or#2 | 229096 |
| #4 | **"High-Intensity Interval Training"[Mesh]** | 2597 |
| #5 | **((((Muscle training[Title/Abstract])) OR (Functional restoration[Title/Abstract])) OR (High Intensity Interval Training[Title/Abstract])) OR (Resistance training[Title/Abstract])** | 22441 |
| #6 | #4or#5 | 23240 |
| #7 | **"Randomized Controlled Trial" [Publication Type]** | 630086 |
| #8 | **((Randomized[Title/Abstract]) OR (placebo[Title/Abstract])) OR (RCT[Title/Abstract])** | 882751 |
| #9 | #7or#8 | 1110716 |
| #10 | #3and#6and#9 | 114 |

Search Strategy of Web of science

| Search  number | Query | Results |
| --- | --- | --- |
| #1 | TS=("Stroke"OR "cerebral strokes"OR "acute stroke" OR "cerebral embolism"OR "cerebral infarction"OR "ischemic stroke"OR "acute cerebrovascular accident") | 261400 |
| #2 | TS=("High-Intensity Interval Training" OR "muscle training"OR "functional restoration"OR "High-Intensity Interval Training"OR "resistance training") | 17184 |
| #3 | TS=("Randomized Controlled Trial"OR "Randomized"OR "placebo" OR "RCT") | 580469 |
| #4 | #2 AND #1 AND #3 | 184 |

Search Strategy of Embase

| Search  number | Query | Results |
| --- | --- | --- |
| #8 | #3 AND #6 AND #7 | 35 |
| #7 | ('randomized controlled trial':it OR randomized:ti,ab,kw OR placebo:ti,ab,kw) AND [embase]/lim | 1104117 |
| #6 | #4OR#5 | 5352 |
| #5 | (('high-intensity intermittent exercise':ti,ab,kw OR 'high-intensity intermittent training':ti,ab,kw OR 'high-intensity interval exercise':ti,ab,kw OR 'high-intensity interval training':ti,ab,kw OR hiie:ti,ab,kw) AND exercise:ti,ab,kw OR 'hiit':ti,ab,kw OR 'intermittent high-intensity training':ti,ab,kw OR 'interval high-intensity training':ti,ab,kw OR 'high intensity interval training':ti,ab,kw) AND [embase]/lim | 4230 |
| #4 | 'high intensity interval training'/exp | 4963 |
| #3 | #1or#2 | 418622 |
| #2 | (('apoplectic':ti,ab,kw OR 'apoplectic stroke':ti,ab,kw OR 'apoplexia':ti,ab,kw OR 'apoplexy':ti,ab,kw) AND 'cerebral apoplexia':ti,ab,kw OR 'cerebral stroke':ti,ab,kw) AND [embase]/lim | 2333 |
| #1 | ('cerebrovascular accident'/exp OR 'cerebrovascular accident') AND [embase]/lim | 417979 |

Search Strategy of Scopus

| Search  number | Query | Results |
| --- | --- | --- |
| #1 | TITLE-ABS-KEY ( stroke ) OR TITLE-ABS-KEY ( cerebral AND strokes ) OR TITLE-ABS-KEY ( acute AND stroke ) OR TITLE-ABS-KEY ( cerebral AND embolism ) OR TITLE-ABS-KEY ( cerebral AND infarction ) OR TITLE-ABS-KEY ( ischemic AND stroke ) OR TITLE-ABS-KEY ( acute AND cerebrovascular AND accident ) | 649616 |
| #2 | TITLE-ABS-KEY ( high-intensity AND interval AND training ) OR TITLE-ABS-KEY ( muscle AND training ) OR TITLE-ABS-KEY ( functional AND restoration ) OR TITLE-ABS-KEY ( high-intensity AND interval AND training ) OR TITLE-ABS-KEY ( resistance AND training ) | 163209 |
| #3 | TITLE-ABS-KEY ( randomized AND controlled AND trial ) OR TITLE-ABS-KEY ( randomized ) OR TITLE-ABS-KEY ( placebo ) OR TITLE-ABS-KEY ( rct ) | 1697631 |
| #4 | ( TITLE-ABS-KEY ( randomized AND controlled AND trial ) OR TITLE-ABS-KEY ( randomized ) OR TITLE-ABS-KEY ( placebo ) OR TITLE-ABS-KEY ( rct ) ) AND ( TITLE-ABS-KEY ( high-intensity AND interval AND training ) OR TITLE-ABS-KEY ( muscle AND training ) OR TITLE-ABS-KEY ( functional AND restoration ) OR TITLE-ABS-KEY ( high-intensity AND interval AND training ) OR TITLE-ABS-KEY ( resistance AND training ) ) AND ( TITLE-ABS-KEY ( stroke ) OR TITLE-ABS-KEY ( cerebral AND strokes ) OR TITLE-ABS-KEY ( acute AND stroke ) OR TITLE-ABS-KEY ( cerebral AND embolism ) OR TITLE-ABS-KEY ( cerebral AND infarction ) OR TITLE-ABS-KEY ( ischemic AND stroke ) OR TITLE-ABS-KEY ( acute AND cerebrovascular AND accident ) ) | 1723 |

Search Strategy of Ovid

| Search  number | Query | Results |
| --- | --- | --- |
| #1 | (Stroke or cerebral strokes or acute stroke or cerebral embolism or cerebral infarction or ischemic stroke or acute cerebrovascular accident).ti,ab,kw | 167233 |
| #2 | (High-Intensity Interval Training or muscle training or functional restoration or High-Intensity Interval Training or resistance training).ti,ab,kw. | 9153 |
| #3 | (Randomized Controlled Trial or Randomized or placebo or RCT).ti,ab,kw. | 345167 |
| #4 | #1 and #2 and #3 | 57 |
