# Supplementary material for: The safety and efficacy of high-intensity interval training (HIIT) in post-stroke patients with moderate functional impairment: a systematic review and meta-analysis
Source: Front Neurol. 2025 Nov 19;16:1695243. doi: 10.3389/fneur.2025.1695243 (PMC12675936; doi:10.3389/fneur.2025.1695243)
Supplement: Supplementary file 2 [file Data_Sheet_2.docx]

**Appendix 2**

The Appendix 2 provides supplementary tables containing additional data visualizations relevant to this study. All tables are numerically indexed and referenced in the main text as appropriate.

Table 1. Characteristics of included studies

| Study | sample size(exp/con)（exp/con）  References:  [1]. Wu, C., et al., Prevalence and Impact of Aphasia among Patients Admitted with Acute Ischemic Stroke. J Stroke Cerebrovasc Dis, 2020. 29(5): p. 104764.  [2]. Gronberg, A., et al., Incidence of Aphasia in Ischemic Stroke. Neuroepidemiology, 2022. 56(3): p. 174-182.  [3]. Helgerud, J., et al., Aerobic high-intensity intervals improve VO2max more than moderate training. Med Sci Sports Exerc, 2007. 39(4): p. 665-71.  [4]. Ross, R., et al., Importance of Assessing Cardiorespiratory Fitness in Clinical Practice: A Case for Fitness as a Clinical Vital Sign: A Scientific Statement From the American Heart Association. Circulation, 2016. 134(24): p. e653-e699.  [5]. Woodruffe, S., et al., Australian Cardiovascular Health and Rehabilitation Association (ACRA) core components of cardiovascular disease secondary prevention and cardiac rehabilitation 2014. Heart Lung Circ, 2015. 24(5): p. 430-41.  [6]. Hsu, C., et al., Increased serum brain-derived neurotrophic factor with high-intensity interval training in stroke patients: A randomized controlled trial. Ann Phys Rehabil Med, 2021. 64(4): p. 101385.  [7]. Hornby, T.G., et al., Cost-Effectiveness of High-intensity Training vs Conventional Therapy for Individuals With Subacute Stroke. Arch Phys Med Rehabil, 2022. 103(7S): p. S197-S204.  [8]. Aidar, F.J., et al., A Randomized Trial Investigating the Influence of Strength Training on Quality of Life in Ischemic Stroke. Top Stroke Rehabil, 2016. 23(2): p. 84-9.  [9]. Anjos, J.M., et al., The impact Of high-intensity interval training On functioning And health-related quality Of life In post-stroke patients: A systematic review With meta-analysis. Clin Rehabil, 2022. 36(6): p. 726-739.  [10]. Tiozzo, E., et al., Aerobic, Resistance, and Cognitive Exercise Training Poststroke. Stroke, 2015. 46(7): p. 2012-6.  [11]. Mayo, N.E., et al., Disablement following stroke. Disabil Rehabil, 1999. 21(5-6): p. 258-68.  capacity, depression and quality of life in patients with coronary artery disease enrolled in cardiac rehabilitation: A randomized controlled trial (CRX study). Prog Cardiovasc Dis, 2022. 70: p. 73-83.  [32]. Mayo, N.E., et al., Disablement following stroke. Disabil Rehabil, 1999. 21(5-6): p. 258-68.  [33]. Flansbjer, U., et al., Reliability of gait performance tests in men and women with hemiparesis after stroke. J Rehabil Med, 2005. 37(2): p. 75-82.  [34]. Michael, K. and R.F. Macko, Ambulatory activity intensity profiles, fitness, and fatigue in chronic stroke. Top Stroke Rehabil, 2007. 14(2): p. 5-12.  [35]. Marzolini, S., et al., Effect of High-Intensity Interval Training and Moderate-Intensity Continuous Training in People With Poststroke Gait Dysfunction: A Randomized Clinical Trial. J Am Heart Assoc, 2023. 12(22): p. e031532.  [36]. Miller, A., et al., Moderate-intensity exercise versus high-intensity interval training to recover walking post-stroke: protocol for a randomized controlled trial. Trials, 2021. 22(1): p. 457.  n） | mean age(exp/con) | Disease duration（exp/con） | | intervention/control | exercise intensity | Course of treatment  （exp/con） | outcomes | follow-up duration |
| --- | --- | --- | --- | --- | --- | --- | --- | --- | --- |
| Lapointe T.(2023) | 15/12 | 71.8±9.9/69.6±10.7 | | 39.3±61.0 mo | HIIT/routine care | 95% of PPO | 24weeks | SBP/HAD/ Peak VO_2_  /PPO | 48weeks |
| Hsu, C. C.(2021) | 10/13 | 58.5±12.16/53.1±9.65 | | 38.5±27.12/  28.8±42.01 mo | HIIT/MICT | 80% of peak VO₂ | 12weeks | Peak VO_2_/AV O2diff/Δ[HHb]/BDNF | no mentioned |
| Hornby T. G.(2024) | 17/11 | 57±9.72/66±11.9 | | 42±45.71/  20±19.72 mo | HIIT(AIT+HIT)/HIT+normoxia | 75% of HRR | 5weeks | Peak VO_2_  /SSS/FS/6MWT | no mentioned |
| Yu C. S.(2022) | 14/16 | 61.86±11.33/46.94±15.61 | | 511.07±292.91/  475.31±411.42 days | HIIT(exowalk60min)/exowalk30min | No mentioned | 2weeks/4weeks | FAC/6MWT | no mentioned |
| Lee Mi-Joung(2008) | 12/12/12/12 | 60.5±10.6/65.3±6 | | 57.0±54.2 mo | HIIT(Cycling)/sham cycling+sham PRT | 70% of peak VO₂ | 12weeks | Peak VO_2_  /PPO/6MWT | no mentioned |
| Hornby T. G.(2022) | 12/17/15 | 52±13/57±12 | | 3.2±1.8/  3.7±1.8 mo | HIIT/routine care | 70-80% of HRR | 10weeks | SF-36/SSS | 8-12weeks |
| Aidar, F. J.(2016) | 11/11 | 51.7±8/52.5±7.7 | | No mentioned | HIIT/routin | 70-80% of HRR | 12weeks | SF-36 | no mentioned |
| Boyne Pierce(2023) | 27/28 | 63.8±9.9/61.5±9.9 | | 2.7±1.4/  2.2±1.2 year | HIIT/MAT | 60% of HRR | 12weeks | VO_2_max/SSS/FS/6MWT | 12weeks |
| Junghwa Do(2024) | 11/11 | 61.8±7.3/63.5±8.1 | | 88.0±81.5/  81.5±52.4 mo | HIIT(RATW)/control | 70% of HRR | 8weeks | VO_2_max/10MWT/FAC | 8weeks |
| Kevin Moncion(2024) | 42/40 | 65.4±8.9/64.4±9.7 | | 1.9±1.3/  1.7±1.3 year | HIIT/MICT | 70-80% of HRR | 20weeks | VO_2_max/6MWT/SBP | 8weeks |
| **VO_2_max:** maximal oxygen consumption, **Peak VO2:** peak oxygen uptake, **HRR:** Heart Rate Reserve, **SBP:** systolic blood pressure, **HAD:** hospital anxiety and depression scale, **PPO:** peak power output, **AVO2diff:** arteriovenous O2 difference, **BDNF:** brain-derived neurotrophic factor, **Δ[HHb]:** deoxyhemoglobin, **SSS:** self-selected speed, **FS:** fastest speed, **6MWT:** 6-minute walk test, **SF-36:** Medical Outcomes Short Form -36 questions, **10MWT:** 10-meter walk test, **FAC:** functional ambulatory catego | | | | | | | | | |

Table 2. Summary of operational definitions for high-intensity and moderate/low-intensity training in the included studies

| Study | HIIT Intensity Definition | | Control Intensity Definition |
| --- | --- | --- | --- |
| Lapointe T.(2023) | 95% of PPO interspersed with a 60-s recovery | usual care without any additional physical activity | |
| Hsu, C. C.(2021) | 80% of peak VO₂  low-intensity recovery periods at 40% VO2peak | 60% of peak VO₂ | |
| Hornby T. G.(2024) | 75% of HRR | Constant oxygen exposure | |
| Yu C. S.(2022) | Exowalk/60min | Exowalk/30min | |
| Lee Mi-Joung(2008) | 70% of peak VO₂ | sham cycling | |
| Hornby T. G.(2022) | 70-80% of HRR | 30-40% of HRR | |
| Aidar, F. J.(2016) | Structured Strength Training | No strength training | |
| Boyne Pierce(2023) | 60% of HRR | 40%±5%of HRR | |
| Junghwa Do(2024) | 70% of HRR | Routine Care | |
| Kevin Moncion(2024) | 80% of HRR | 40-59% of HRR | |
|  | | | |

Table 4. Summary of Safety and Compliance for Interventions

| Study | Total Participants (n) | Adverse events in the intervention group | Adverse events in the control group | Overall shedding rate(exp/con) | Primary causes of shedding | Compliance (%) |
| --- | --- | --- | --- | --- | --- | --- |
| Lapointe T.(2023) | 36 | No serious adverse events | No serious adverse events | 21%/29% | Lack of interest (n=6)  Change in medical condition (n=3) | HIIT group (77%) |
| Hsu, C. C.(2021) | 28 | No serious adverse events | No serious adverse events | 23%/13.3% | Recurrent stroke (n=2)  Unstable BP (n=1)  Hernia suegery (n=1)  Incomplete ex. (n=1) | No mentioned |
| Hornby T. G.(2024) | 35 | One serious adverse event was observed following AIH exposure | No serious adverse events | 15%/26.7% | Personal reasons (n=5)Taking banned substances (n=1)Dizziness reaction (n=1)Personal reasons | No mentioned |
| Yu C. S.(2022) | 36 | No serious adverse events | No serious adverse events | 22.2%/11.1% | FAC was level 1 (n=2)  FAC was level 6 (n=4) | No mentioned |
| Lee Mi-Joung(2008) | 25 | No serious adverse events | No serious adverse events | 7.6%/0% | changes in their health status (n=1) | No mentioned |
| Hornby T. G.(2022) | 29 | No serious adverse events | No serious adverse events | 0%/0% | No detachment has occurred. | No mentioned |
| Aidar, F. J.(2016) | 27 | Three patients dropped out due to personal circumstances. | Two patients dropped out due to personal circumstances. | 0%/15.3% | Personal reasons (n=5) | HIIT group (94%)  Control group (No mentioned  ) |
| Boyne Pierce(2023) | 55 | No serious adverse events | No serious adverse events | 30%/18% | Participants voluntarily withdrew (n=7) Back pain (n=1) Recurrent hamstring strain (n=1) COVID-19 (n=4) | HIIT group (82.3%)  Control group (86.8%) |
| Junghwa Do(2024) | 24 | No serious adverse events | No serious adverse events | 8.3%/8.3% | Unknown reason (n=2) | No mentioned |
| Kevin Moncion(2024) | 82 | No serious adverse events | No serious adverse events | 21.4%/32,5% | Medical conditions (n=9)  COVID-19 (n=6)  Transportation issues (n=2)  Return to work (n=2)  Other rehabilitation (n=1)  Preference against intervention plan (n=1)  Unknown reasons (n=1)  Missing (n= 12) | HIIT group (99%)  Control group (99%) |

Table 3. Quality assessment and risk of bias

| Study | Random-ization process | allocation process | blinding deficiencies | assessment of outcomes | follow-up | reporting of results | Other bias |
| --- | --- | --- | --- | --- | --- | --- | --- |
| Lapointe T.  (2023) | Low | Low | High | Low | Low | Unclear | Unclear |
| Hsu, C.C.  (2021) | Low | Low | Low | Low | Low | Unclear | Unclear |
| Hornby T.G.  (2024) | Low | Low | Low | Low | Low | Unclear | Unclear |
| Yu C. S.  (2022) | Low | Low | Unclear | Low | Low | Unclear | Unclear |
| Lee Mi-Joung  (2008) | Low | Unclear | Unclear | Low | Low | Unclear | Unclear |
| Hornby T.G.  (2022) | Low | Unclear | Unclear | Low | Low | Low | Unclear |
| Aidar, F.J.  (2016) | Unclear | Unclear | High | Unclear | Low | Low | Unclear |
| Boyne Pierce  (2023) | Low | Low | Low | Low | Low | Unclear | Unclear |
| Junghwa Do  (2024) | Unclear | Unclear | High | Low | Low | Unclear | Unclear |
| Kevin Moncion  (2024) | Low | Low | Low | Unclear | Low | Unclear | Unclear |
